# The sucrose–trehalose 6-phosphate nexus is conserved in flowering plants with different phloem loading and carbon storage strategies

**DOI:** 10.1093/jxb/eraf401

**Published:** 2025-10-23

**Authors:** Maria G Annunziata, Regina Feil, Marc Lohse, Carlos M Figueroa, Matías D Hartman, Mohammad Esmailpour, Zoran Nikoloski, Karin Koehl, Mark Stitt, John E Lunn, Franziska Fichtner

**Affiliations:** Max Planck Institute of Molecular Plant Physiology, Am Muehlenberg 1, Potsdam 14476, Germany; Max Planck Institute of Molecular Plant Physiology, Am Muehlenberg 1, Potsdam 14476, Germany; Max Planck Institute of Molecular Plant Physiology, Am Muehlenberg 1, Potsdam 14476, Germany; Max Planck Institute of Molecular Plant Physiology, Am Muehlenberg 1, Potsdam 14476, Germany; Instituto de Agrobiotecnología del Litoral, UNL, CONICET, FBCB, Colectora Ruta Nacional 168km 0, Santa Fe, Argentina; Instituto de Agrobiotecnología del Litoral, UNL, CONICET, FBCB, Colectora Ruta Nacional 168km 0, Santa Fe, Argentina; Max Planck Institute of Molecular Plant Physiology, Am Muehlenberg 1, Potsdam 14476, Germany; Max Planck Institute of Molecular Plant Physiology, Am Muehlenberg 1, Potsdam 14476, Germany; Bioinformatics Department, Institute of Biochemistry and Biology, University of Potsdam, Potsdam 14476, Germany; Max Planck Institute of Molecular Plant Physiology, Am Muehlenberg 1, Potsdam 14476, Germany; Max Planck Institute of Molecular Plant Physiology, Am Muehlenberg 1, Potsdam 14476, Germany; Max Planck Institute of Molecular Plant Physiology, Am Muehlenberg 1, Potsdam 14476, Germany; Heinrich Heine University Düsseldorf, Faculty of Mathematics and Natural Sciences, Institute of Plant Biochemistry, and Cluster of Excellence on Plant Sciences (CEPLAS), Düsseldorf 40225, Germany; The James Hutton Institute, UK

**Keywords:** Apoplastic phloem loading, phloem, primary metabolism, sucrose, symplastic phloem loading, trehalose 6-phosphate

## Abstract

Trehalose 6-phosphate (Tre6P) is a signalling metabolite that maintains sucrose homeostasis and links plant growth and development to the availability of sucrose. Most of our knowledge of the nexus between Tre6P and sucrose comes from studies on arabidopsis (*Arabidopsis thaliana*), and it is unclear whether this close relationship is generally conserved across other species. To address this question, we investigated the diel changes in sucrose and Tre6P in leaves from a phylogenetically diverse set of angiosperms with different phloem loading and carbohydrate storage strategies: arabidopsis, *Alchemilla molis*, strawberry (*Fragaria×ananassa*), *Plantago major*, melon (*Cucumis melo*), and wheat (*Triticum aestivum*). Despite large differences in their sucrose and Tre6P levels, there were positive correlations between sucrose and Tre6P across all species. Network analysis confirmed a strong association between Tre6P and sucrose in all species, and also revealed a common link with malate, consistent with positive regulation of malate synthesis by Tre6P. In combination with previous observations that Tre6P is synthesized in and around the leaf vasculature, our findings suggest that Tre6P primarily reflects the vascular transport pool of sucrose in leaves. We conclude that the sucrose–Tre6P nexus is widespread among angiosperms, with a conserved role in regulation of sucrose metabolism and transport.

## Teaser

Trehalose 6-phosphate (Tre6P) has a homeostatic relationship with sucrose in the model plant arabidopsis. This Tre6P–sucrose relationship is conserved across diverse flowering plants, suggesting a universal sucrose sensing mechanism.

## Introduction

Plants assimilate CO_2_ in their leaves during the daytime and synthesize carbohydrates for storage and export to non-photosynthetic tissues to supply the latter with carbon and energy. Typically, up to 80% of the fixed carbon is exported from source leaves in the form of sugars to non-photosynthetic sink organs via long-distance transport through the phloem (Ayre and Turgeon, 2018). In most plants this is achieved by transporting the non-reducing disaccharide sucrose from source to sink tissues (Stitt *et al*., 2010; Lunn, 2016). To provide the plant with energy during the night-time and to buffer unpredictable periods of low carbon availability (Annunziata *et al*., 2017, 2018), plants have evolved mechanisms to store some of the assimilated carbohydrates. Many plants, including arabidopsis (*Arabidopsis thaliana*), accumulate large amounts of starch in their leaves during the daytime, which are then degraded during the night to supply carbon for maintenance respiration and growth (Stitt and Zeeman, 2012). However, starch is not the only transitory storage compound in leaves. In addition to starch, many plants accumulate sucrose, other soluble sugars, or fructans, which are stored mainly in vacuoles (Lunn, 2016; Braun, 2022). In members of the *Poaceae*, such as wheat (*Triticum aestivum*), sucrose is often the predominant form of stored carbohydrate (Lunn and Furbank, 1997; Castleden *et al*., 2004).

Since the appearance of vascular transport systems in the *Tracheophyta*, plants have evolved different phloem loading strategies, which can be broadly divided into symplastic and apoplastic types. Passive symplastic phloem loading occurs via passive diffusion of sucrose from mesophyll cells into the phloem through plasmodesmata, requiring high sucrose concentrations throughout the symplast to generate hydrostatic pressure to drive solute flow through the phloem sieve elements (Turgeon, 2010; Ayre and Turgeon, 2018; Braun, 2022). This is probably the ancestral form of phloem loading, and is common in tropical rainforest trees and shrubs, such as *Amborella trichopoda* and other basal angiosperms that are thought to represent the earliest stages in angiosperm evolution. However, passive symplastic phloem loading is also found in some eudicots, including herbaceous members of the *Rosaceae* family, such as alchemilla (*Alchemilla mollis*) and strawberry (*Fragaria×ananassa*).

A specialized form of symplastic phloem loading, known as ‘polymer trapping’, is mainly found in species from the *Cucurbitaceae* such as melon (*Cucumis melo*). In these plants, sucrose diffuses passively from the mesophyll into specialized companion cells, known as intermediary cells, where it is converted into larger raffinose family oligosaccharides (RFOs), such as raffinose or stachyose, by addition of galactose moieties (Beebe and Turgeon, 1992). These larger molecules are now ‘trapped’ within the companion cell–sieve element complex as the plasmodesmata are too small to allow diffusion of these larger molecules back into mesophyll cells (Botha and Murugan, 2021). The accumulation of RFOs increases the osmotic potential in the intermediary cells, drawing water into the phloem and generating a hydrostatic pressure gradient that facilitates long-distance transport to sink tissues (Turgeon, 2010). Due to the energy required for synthesis of RFOs from sucrose, this is considered to be an active symplastic phloem loading mechanism.

In apoplastic phloem loaders like arabidopsis and wheat, sucrose synthesized in mesophyll cells is initially transported symplastically through plasmodesmata to the phloem parenchyma cells within the vascular bundles. Sucrose is then exported from these cells into the apoplast via SUGARS WILL EVENTUALLY BE EXPORTED TRANSPORTER (SWEET) type transporters in the plasmalemma (Chen *et al*., 2012), and subsequently taken up into the companion cell–sieve element complex via SUT-type sucrose–H^+^ symporters (Lalonde *et al*., 2003; Slewinski *et al*., 2009). This active loading results in high sucrose concentrations within the sieve elements, drawing water in osmotically to create a hydrostatic pressure gradient that drives sucrose transport to sink tissues (Knoblauch *et al*., 2016).

In addition to sucrose and RFOs, sugar alcohols represent another class of phloem-transported solutes. Some members of the *Rosaceae* (e.g. apple trees) or *Plantaginaceae*, such as plantago (*Plantago major*), transport both sucrose and sugar alcohols like sorbitol (Reidel *et al*., 2009). Depending on the species, these solutes are loaded via symplastic (e.g. apple) or apoplastic (e.g. plantago) pathways (Reidel *et al*., 2009).

Although sucrose may share its role as a transport sugar with RFOs or sugar alcohols in some species, it is by far the most commonly transported sugar in angiosperms and is never completely displaced in this role. Similarly, even in species that transiently store mostly starch or other carbohydrates in their leaves, sucrose is always a major product of photosynthesis. Sucrose is a non-reducing disaccharide that is chemically stable, highly soluble, and able to stabilize protein and membrane structures, and these properties could be the key to its roles as a transport sugar and osmolyte in plants (Pontis, 1977).

The only other non-reducing disaccharide that is commonly found in nature is α,α-trehalose (trehalose), which fulfils similar functions to sucrose in fungi, insects and some other invertebrates. Apart from some desiccation-tolerant resurrection plants, vascular plants usually contain only trace amounts of trehalose (Carillo *et al*., 2013; Lunn *et al*., 2014). However, the phosphorylated intermediate of trehalose biosynthesis, trehalose 6-phosphate (Tre6P), has been shown to be essential for normal growth and development in arabidopsis, maize (*Zea mays*), and other plants (Eastmond *et al*., 2002; Schluepmann *et al*., 2003; Satoh-Nagasawa *et al*., 2006; Wahl *et al*., 2013; Claeys *et al*., 2019; Fichtner *et al*., 2021a, b; Klein *et al*., 2022), with a role in sucrose signalling and homeostasis (Lunn *et al*., 2006; Yadav *et al*., 2014; Figueroa *et al*., 2016; Miret *et al*., 2024). In source leaves, a major role of Tre6P is sucrose homeostasis, i.e. regulating the production and export of sucrose to balance sucrose supply with demand from sink organs. In arabidopsis leaves in the light, Tre6P regulates photoassimilate partitioning between sucrose and organic and amino acids, via post-translational regulation of phospho*enol*pyruvate carboxylase (PEPC) and nitrate reductase (Figueroa *et al*., 2016). At night, Tre6P regulates the production of sucrose from turnover of transitory starch (Martins *et al*., 2013; dos Anjos *et al*., 2018; Ishihara *et al*., 2022). In sink organs, which depend on sucrose for most of their carbon and energy needs, Tre6P signals the availability of sucrose to regulate growth accordingly, in part via regulation of SUCROSE-NON-FERMENTING-1-RELATED PROTEIN KINASE 1 (SnRK1) (Zhang *et al*., 2009; Zhai *et al*., 2018; Baena-González and Lunn, 2020; Avidan *et al*., 2024; Lopes *et al*., 2024). In meristematic tissues, Tre6P signalling is integrated with phytohormone signalling to control developmental decisions, such as flowering and shoot branching, that commit the plant to future growth and increased demand for sucrose (reviewed in Fichtner and Lunn, 2021; Göbel and Fichtner, 2023). Once the plant is committed to new growth, Tre6P also plays a role in coordinating central carbon and nitrogen metabolism to provide the building blocks for growth (Fichtner *et al*., 2017). The dual nature of Tre6P as both a signal and homeostatic regulator of sucrose is sometimes referred to as the sucrose–Tre6P nexus (Yadav *et al*., 2014; Figueroa and Lunn, 2016).

In arabidopsis, TREHALOSE-6-PHOSPHATE SYNTHASE1 (TPS1) is solely responsible for Tre6P synthesis in leaves, and is located mainly in the vasculature, including xylem and phloem parenchyma cells and the companion cell–sieve element complex (Fichtner *et al*., 2020; Fichtner and Lunn, 2021). Arabidopsis is predominantly an apoplastic phloem loader. The phloem parenchyma is symplastically connected with the mesophyll cells and is largely responsible for export of sucrose into the apoplast, from where it is actively taken up by the companion cells into the phloem. Thus, the cell types where TPS1 is located represent the interface between source and sink tissues. Tre6P is therefore produced at a highly strategic site for systemic sensing and signalling of sucrose status (Fichtner *et al*., 2020; Fichtner and Lunn, 2021). Transcript profiling studies suggest that *AtTPS1* orthologues are predominantly expressed in the vascular tissue in leaves of other species, including wheat (Zhang *et al*., 2023) and plantago (Huang *et al*., 2019), both of which are apoplastic phloem loaders, and in *Prunus mume* (Guo *et al*., 2024), a member of the *Rosaceae* that is likely to be a symplastic phloem loader that transports sucrose and sorbitol (Rennie and Turgeon, 2009). These findings point to Tre6P signalling being associated with the vascular tissue and phloem loading across diverse species. Nevertheless, given the diversity of carbohydrate metabolism in angiosperms, caution is warranted in extrapolating our knowledge of Tre6P signalling, gained mainly from studies of arabidopsis, to other species.

In this study, we analysed diel changes in leaf metabolism across six angiosperms with different phloem loading and carbon storage strategies—arabidopsis, wheat, melon, alchemilla, strawberry and plantago (Supplementary Fig. S1)—to test whether the arabidopsis sucrose–Tre6P nexus model is relevant for other species. We also measured a broad range of other primary metabolites and used hierarchical clustering and network analyses to reveal other metabolites with links to Tre6P. We also used these approaches to explore associations between metabolites within these diverse species. We identified some features that are conserved across the species, but also some differences that could give insights into how central metabolism is adapted in species with different phloem loading and carbon storage strategies. We found positive correlations between Tre6P and sucrose in all of the species, and a frequent connection with malate. Our findings suggest that Tre6P is strongly linked to the transport pool of sucrose in leaves and that this association is conserved across species, irrespective of their phloem loading strategy.

## Material and methods

### Plant growth and harvest

*Arabidopsis thaliana* [L.] Heynh accession Columbia 0 (Col-0) seeds were obtained from the MPI-MP in-house collection. Seeds were sown on soil (Stender, Schermbeck, Germany) mixed with vermiculite (1:1) soaked with tap water, supplemented with boron (1.8 mg L^–1^) and the fungicide Previcur (1.5 ml L^–1^; Bayer). At 14 days after sowing (DAS) plants were transplanted to 10 cm-diameter pots (five plants per pot) of soil:vermiculite (1:1) and transferred to equinoctial conditions (12 h photoperiod) in a Percival Scientific AR-75L cabinet (Percival Scientific) with white fluorescent tubes giving a constant irradiance of 160 µmol m^−2^ s^−1^. Day/night temperatures were 20 °C/18 °C with a relative humidity (RH) of 65%. Rosettes were harvested from 28-day-old plants at 2 h intervals during a complete 24 h light–dark cycle, with additional sampling at 0.5–1 h intervals around dawn and dusk. Rosettes were harvested under ambient growth conditions and immediately quenched in liquid nitrogen, and rosettes from five plants were pooled for each biological replicate.

*Triticum aestivum* L. cv. Hamon was grown in 20 cm pots containing a mixture of soil, perlite, sand, and peat moss (50:25:15:10 by volume) and Osmocote slow-release fertilizer. The plants were illuminated with metal halide lamps providing a photosynthetic photon flux density of 500 μmol m^−2^ s^−1^ at the leaf surface, with a 14 h photoperiod. The day and night temperatures were 25 °C and 22 °C, respectively, and a constant RH of 65% was maintained. Each biological replicate consists of two to three fully expanded leaves from individual 6-week-old wheat plants harvested at 0.5–1 h intervals around dawn and dusk and at 2 h intervals during the rest of the diurnal cycle.

*Alchemilla mollis* [Buser] Rothm. and *Fragaria×ananassa* Duchesne seeds were obtained from a commercial garden supplier, sown on soil mixed with vermiculite (1:1) and germinated in a controlled environment chamber under short day conditions (8 h photoperiod) with an irradiance of 100 µmol m^−2^ s^−1^ provided by white fluorescent lamps. The day/night temperatures were 20 °C/16 °C, and RH was 60% during the day and 75% at night. At 7 DAS, the light regime was switched to long day conditions (16 h photoperiod), with all other environmental conditions unchanged. At 14 DAS seedlings were transplanted to 6×6 cm square pots (one plant per pot) of soil:vermiculite (1:1) and transferred to a Percival Scientific AR-75L cabinet under long day conditions with white fluorescent tubes giving a constant irradiance of 160 µmol m^−2^ s^−1^. Day/night temperatures were 20 °C/18 °C with a RH of 65/75%. At 70 DAS, leaf samples were harvested at 2 h intervals during a complete 24 h light–dark cycle, with additional sampling at 0.5–1 h intervals around dawn and dusk. For each biological replicate two fully expanded leaves (without the petiole) were harvested and immediately frozen in liquid nitrogen.

*Cucumis melo* L. cv. Vedrantais seeds were obtained from Dr Jordi Garcia Mas (IRTA-CRAG, Barcelona, Spain) and germinated on moist filter paper. Two days after germination, seeds were placed into 6×6 cm square pots (one seed per pot) of a low-nutrient peat-based substrate (Kausek, Mittenwalde, Germany) and transferred to a controlled-environment chamber under long day conditions (16 h photoperiod) with an irradiance of 250 µmol m^−2^ s^−1^, provided by white fluorescent lamps, day/night temperatures of 25 °C/20 °C, and a RH of 65%. At 14 d after germination the young plants were transferred to 13×13 cm square pots (one per pot) filled with peat-based substrate and sand (2:1; Kausek, Mittenwalde, Germany). At 28 d after germination plants were transferred to 30×30 cm square pots (one per pot) and 7 d later supplemented with Plantosan (Hauert Manna Düngerwerke, Nuremburg, Germany) slow-release fertilizer with an N:P:K (Mg) ratio of 20:10:15(6). The plants were moved to a ventilated and naturally illuminated greenhouse (location 52°24′55.2″N, 12°58′5.5″E) with a polyethylene shell without temperature control. At 70 DAS, when the natural daylength was 16.5 h, leaf discs (1 cm diameter) were harvested from fully expanded leaves and 10 discs were pooled per biological sample. Samples were taken at 2–4 h intervals during a complete 24 h light–dark cycle, with additional sampling at 0.5–1 h intervals around dawn and dusk.

*Plantago major* L. (PLA 59, IPK Gatersleben Genebank) plants were grown at the Instituto de Agrobiotecnología del Litoral, Santa Fe, Argentina. Seeds were sown on soil (TS1 substrate; Klasmann-Deilmann, Geeste, Germany) and grown in a controlled environment chamber (Aralab, Portugal) under equinoctial conditions (12 h photoperiod) with white fluorescent tubes providing a constant irradiance of 120 µmol m^−2^ s^−1^, with a day/night temperature of 23 °C and a RH of 70%. At 14 DAS seedlings were transplanted to 8 cm diameter pots (one plant per pot). At 30 DAS samples were harvested at 2 h intervals during a complete 24 h light–dark cycle and immediately frozen in liquid nitrogen. Each biological replicate was composed of leaves 1–4 from the same plant.

All frozen tissues were ground to a fine powder at −70 °C using an automated cryogenic grinding robot (Stitt *et al*., 2007) and stored at −80 °C until analysed.

### Extraction and measurement of metabolites

Soluble sugars (glucose, fructose, and sucrose) were extracted with boiling 80% (v/v) ethanol and assayed enzymatically as described previously (Stitt *et al*., 1989). Starch was determined enzymatically in the insoluble material remaining after the ethanolic extraction (Hendriks *et al*., 2003). The same procedure was used to measure glucose, fructose, sucrose, sorbitol, and starch in plantago samples, with the soluble ethanolic extracts being dried using a centrifugal desiccator and then redissolved in water before analysis. For enzymatic determination of sorbitol, a recombinant sorbitol dehydrogenase from peach was used (Hartman *et al*., 2014). Tre6P, phosphorylated intermediates, and organic acids were extracted with chloroform/methanol, and measured by high-performance anion-exchange liquid chromatography coupled to tandem mass spectrometry (LC-MS/MS) as described in Lunn *et al*. (2006) with published modifications (Figueroa *et al*., 2016). Soluble sugars and oligosaccharides in melon leaf extracts were measured according to Feil and Lunn (2018).

### Statistical analyses

The metabolite levels were normalized by *z*-score prior to use for principal component analysis (PCA) performed using XLStat-Base (Addinsoft, New York, USA). Heatmap analyses were performed on the averages of each time point of the data in Supplementary Table S1 with the heatmap.2 function (R package heatmaply version 1.0.12) using the agglomeration method ‘mcquitty’ for the hierarchical cluster analysis and the distance measure ‘canberra’ for the computation of the distance matrix. Metabolites were clustered using the Pearson’s pairwise correlations as dissimilarity measures. Pearson correlation coefficients and *P*-values were calculated based on data in Supplementary Table S1 using the function rcorr function (R package Hmisc version 5.2-2). The metabolic network analyses were done using the averages of each time point of the data in Supplementary Table S1 and the qgraph function (R package qgraph version 1.9.8). Networks were plotted using the igraph function (R package igraph version 2.1.4) with a Fruchterman Reingold layout. The qgraph function uses the glasso package (Friedman *et al*., 2015) to compute a sparse Gaussian graphical model with the graphical LASSO (Friedman *et al*., 2008). The tuning parameter 0.25 was chosen for all analyses, rather than through cross-validation, to facilitate the comparisons between species. Community detection was done using community structure via greedy optimization of modularity using the cluster_fast_greedy function (R package igraph version 2.1.4) with default settings.

## Results

### The correlation between trehalose 6-phosphate and sucrose is conserved in species with different phloem loading strategies

For the comparative analysis of Tre6P and sucrose, we selected six different species representing different phloem loading strategies: (i) arabidopsis and wheat, both apoplastic phloem loaders that transport sucrose; (ii) plantago, an apoplastic phloem loader that transports both sucrose and sorbitol; (iii) strawberry and alchemilla, passive symplastic phloem loaders that transport sucrose; and (iv) melon, an active symplastic phloem loader that transports sucrose and RFOs. Arabidopsis, wheat, melon, and plantago were cultivated under conditions optimized for their respective growth, based on our previous experience of working with these species. For alchemilla and strawberry we chose similar conditions to those used for arabidopsis, but with a longer photoperiod, and found that the plants grew well under these conditions. Samples of fully expanded source leaves were collected at intervals of 0.5–2 h over a full 24 h light–dark cycle for metabolite analysis (Supplementary Table S1).

**Fig. 1.**
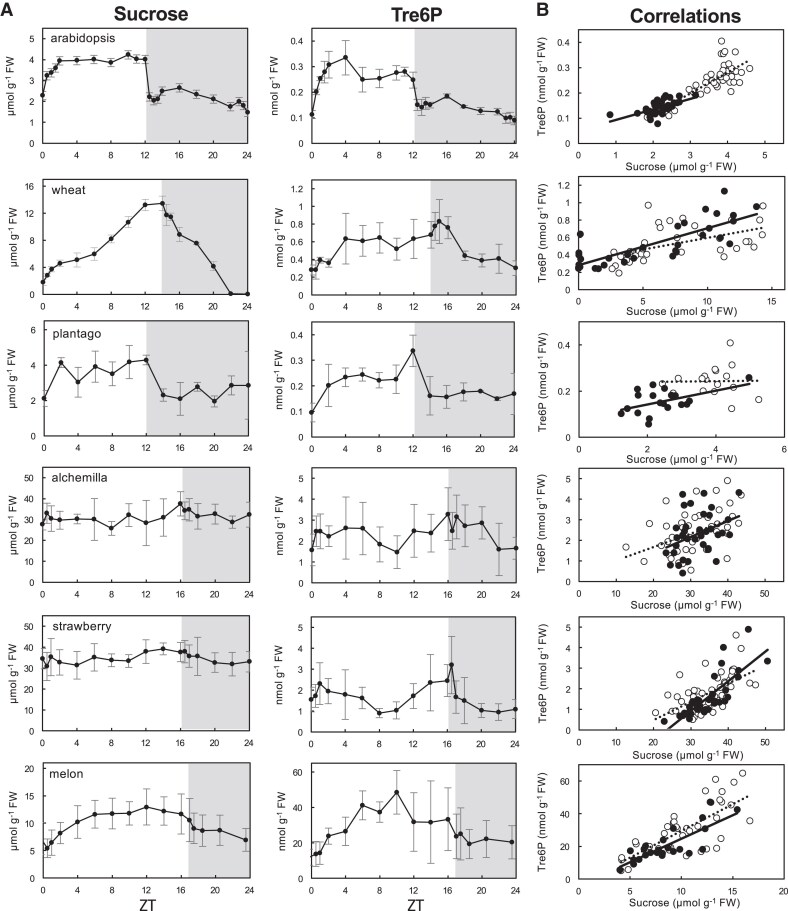
Relationship between sucrose and trehalose 6-phosphate (Tre6P) across six different species with different phloem loading strategies. (A) Diel changes of sucrose and Tre6P. Data points are means ±SD, *n*=3–5. (B) Pearson correlation between sucrose and Tre6P for daytime (open symbols, dashed line) and night-time points (closed symbols, solid line) are shown. Regression lines for daytime (dashed) and night (solid) are given; the regression line of the combined dataset is not shown. FW, fresh weight; ZT, zeitgeber time (time after dawn). Pearson correlations coefficients (*r*) and *P*-values are summarized in Table 1. The original data are in Supplementary Table S1.

To explore the relationship between sucrose and Tre6P, we compared their levels and examined their correlation during the day or night, and across the complete diel cycle (Fig. 1A; Table 1; Supplementary Table S1). Tre6P and sucrose levels varied substantially among the species. Alchemilla and strawberry, which store sucrose and translocate it symplastically, had the highest sucrose levels [approx. 30 µmol g^−1^ fresh weight (FW), Fig. 1A], 5–20 times greater than the other species. They were followed by the symplastic RFO loader melon (approx. 10 µmol g^−1^ FW). Among the apoplastic loaders, sucrose levels were higher in wheat (3–12 µmol g^−1^ FW) than in arabidopsis and plantago (2–4 µmol g^−1^ FW). Melon leaves also contained substantial levels of the RFOs raffinose (0.08–0.6 µmol g^−1^ FW) and stachyose (1–1.7 µmol g^−1^ FW; Supplementary Fig. S2). Plantago leaves also contained high levels of sorbitol (8–18 µmol g^−1^ FW; Supplementary Fig. S2).

**Table 1.** Correlation between trehalose 6-phosphate and phloem-transported sugars in six species with different phloem loading strategies

Species	Sugar	Pearson correlation coefficient (*r*)			*P*-value		
		Day	Night	Diel 24 h	Day	Night	Diel 24 h
Arabidopsis	Sucrose	**0**.**72**	**0**.**73**	**0**.**91**	<4.7×10^−8^	<3.9×10^−9^	0
Wheat	Sucrose	**0**.**53**	**0**.**80**	**0**.**67**	<0.001	<3.2×10^−8^	<4.6×10^−10^
Plantago	Sucrose	0.02	**0**.**48**	**0**.**57**	0.946	<0.034	<0.0003
Plantago	Sorbitol	0.35	0.37	0.07	0.155	0.124	0.699
Alchemilla	Sucrose	**0**.**43**	**0**.**37**	**0**.**40**	<0.002	<0.03	<0.0002
Strawberry	Sucrose	**0**.**59**	**0**.**80**	**0**.**68**	<6.1×10^−6^	<9.9×10^−9^	<1.1×10^−12^
Melon	Sucrose	**0**.**74**	**0**.**81**	**0**.**77**	<6.5×10^−8^	<1.9×10^−6^	<1.3×10^−13^
Melon	Raffinose	**0**.**65**	**0**.**72**	**0**.**70**	<5.2×10^−6^	<6.8×10^−5^	<1.3×10^−10^
Melon	Stachyose	−0.15	−0.12	−0.07	0.370	0.589	0.581

Values for the Pearson correlation coefficient (*r*) and *P*-values were calculated for: (i) samples harvested in the light (day), (ii) samples harvested in the dark (night), and (iii) for all samples harvested during the 24 h light–dark cycle (diel 24 h). *r*-values of significant correlations (*P*<0.05) are shown in bold. Plots of the diel changes and regression plots for the daytime samples and the night-time samples are provided in Fig. 1.

Tre6P levels also differed dramatically between species. Melon had the highest Tre6P levels (20–40 nmol g^−1^ FW, Fig. 1A), 20–100 times greater than the other species. Among the remaining species, alchemilla and strawberry (approx. 2 nmol g^−1^ FW) had higher Tre6P levels than wheat (approx. 0.5 nmol g^−1^ FW), with arabidopsis and plantago showing the lowest levels (0.1–0.3 nmol g^−1^ FW, Fig. 1A).

Across the whole diel cycle, sucrose and Tre6P levels were positively and significantly correlated in all species (Fig. 1B; Table 1). The strongest significant correlation was observed in arabidopsis (*r*=0.91, *P*=0), followed by melon (0.77, *P*<1.3×10^−13^), wheat (0.67, *P*<4.6×10^−10^), strawberry (0.68, *P*<1.1×10^−12^), plantago (0.57, *P*<0.0003), and alchemilla (0.40, *P*<0.0002). When day- and night-harvested samples were analysed separately, sucrose and Tre6P were still significantly correlated in all species except plantago during both phases of the diel cycle. Indeed, the night-time samples from wheat, strawberry, and melon showed even higher correlation coefficients than in the complete diel analysis, and there was a correlation for the night-time samples from plantago, but not for the daytime-only samples from this species (Table 1). There was also no significant correlation between Tre6P and sorbitol in plantago (Table 1). In melon, we observed a significant positive correlation between Tre6P and raffinose during the whole diel cycle (*r*=0.70, *P*<1.3×10^−10^), and when the day and night samples were analysed separately, although these correlations were all slightly weaker than the corresponding correlations between Tre6P and sucrose (Table 1). There was no correlation between Tre6P and stachyose (Table 1).

There were marked differences between species in their Tre6P:sucrose ratios (Supplementary Fig. S3). Melon had the highest ratio with a median value of 2.77 nmol/µmol^−1^. All the other species had much lower Tre6P:sucrose ratios with the following median values: wheat, 0.09 nmol/µmol; alchemilla, 0.07 nmol/µmol; arabidopsis, 0.06 nmol/µmol; plantago, 0.06 nmol/µmol; and strawberry 0.04 nmol/µmol. There were no obvious trends separating the apoplastic and symplastic phloem loading species.

### Principal component analysis and metabolite clustering distinguish phloem loading strategies across species

**Fig. 2.**
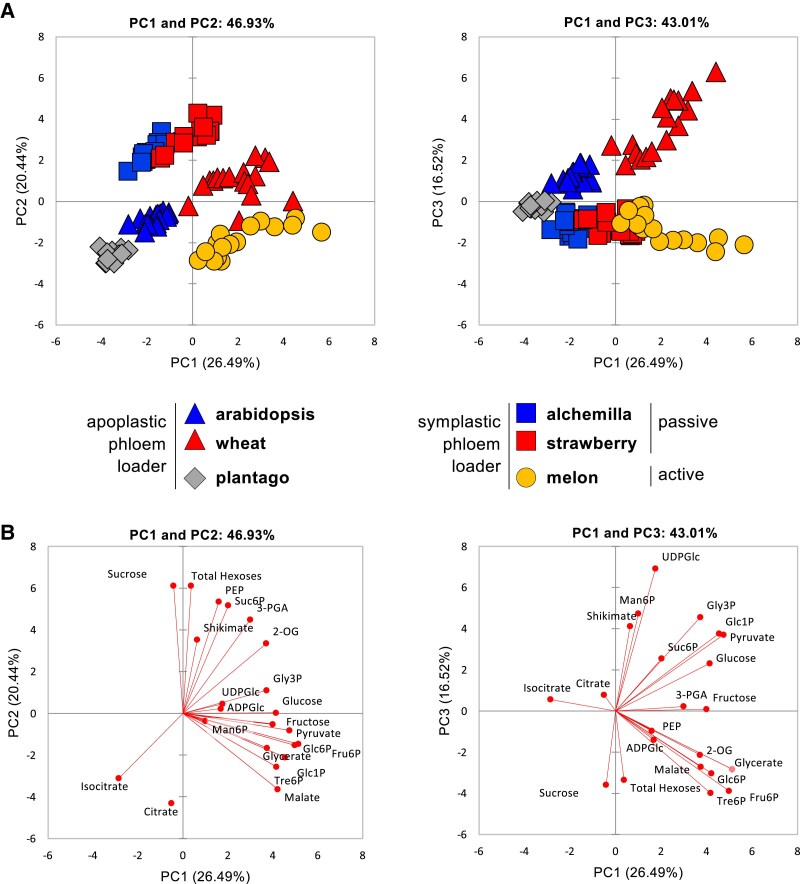
Principal component analysis (PCA) of metabolite data from all datasets. (A) PCA of metabolite data from arabidopsis (blue triangles) and plantago (grey diamonds) plants grown in controlled environment chambers with a 12 h photoperiod, wheat (red triangles), alchemilla (blue squares), and strawberry (red squares) plants grown in controlled environment chambers with a 14, 16, and 16 h photoperiod, respectively, and melon (orange circles) plants grown in a naturally illuminated greenhouse in a 16.5 h natural photoperiod. The percentages of total variance represented by principal component 1 (PC1), PC2, and PC3 are shown in parentheses. (B) Loadings of individual metabolites (red) on the principal components. Only metabolites measured in all datasets were included in the PCAs after *z*-score normalization. 2-OG, 2-oxoglutarate; 3-PGA, 3-phosphoglycerate; ADPGlc, adenosine 5′-diphosphoglucose; Fru6P, fructose 6-phosphate; Glc1P, glucose 1-phosphate; Glc6P, glucose 6-phosphate; Gly3P, glycerine 3-phosphate; Man6P, mannose 6-phosphate; PEP, phospho*enol*pyruvate; Suc6P, sucrose 6′-phosphate; Tre6P, trehalose 6-phosphate; UDPGlc, uridine 5′-diphosphoglucose.

To further explore interspecies metabolic variation, we performed PCA on *z*-score-normalized metabolic profiles using a core dataset of metabolites measured in all species (Fig. 2A). PC1 captured the greatest variance (26%), followed by PC2 (20%) and PC3 (17%). Discrimination between phloem loading strategies required all three principal components. While PC1 alone did not separate species based on phloem loading type, PC2 effectively distinguished symplastic passive loaders (alchemilla and strawberry), apoplastic loaders (arabidopsis, plantago, wheat), and the active symplastic loader (melon), particularly when combined with PC1 (Fig. 2A). PC3 further differentiated apoplastic from symplastic loaders, independently of whether the latter used passive or active loading (Fig. 2A, right panel). No clear separation was observed between daytime and night-time samples (Supplementary Fig. S4).

Separation along PC2 was primarily driven by positive loadings for sucrose, hexoses, sucrose 6^F^-phosphate (Suc6P), shikimate, 3-phosphoglycerate (3-PGA), and 2-oxoglutarate (2-OG), and negative loadings for several organic acids (citrate, isocitrate, malate), Tre6P, and hexose-phosphates [glucose 1-phosphate (Glc1P), glucose 6-phosphate (Glc6P), fructose 6-phosphate (Fru6P)] (Fig. 2B). PC3 captured similar metabolite drivers but with reversed loading directions for some compounds: sucrose and hexoses had negative loadings, while citrate and isocitrate had positive loadings. Additionally, PC3 showed strong contributions from uridine 5′-diphosphoglucose and mannose 6-phosphate (Man6P).

### Clustering and pairwise correlation analysis reveals similarities and differences in metabolite profiles between species

**Fig. 3.**
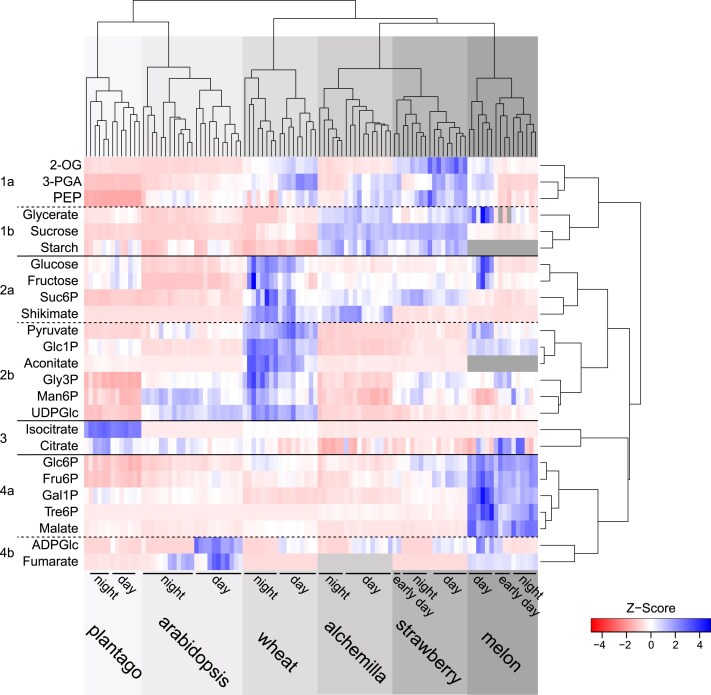
Cross-species comparison of metabolite levels over the entire diel cycle via clustering analysis of all common metabolites. *z*-scores of the mean are presented for each individual metabolite, with high and low *z*-scores shown in blue or red, respectively. Numbers, dashed and solid lines highlight clusters among metabolites. Dendrograms represent clusters based on a Canberra distance matrix with mcquitty-based clustering of species and correlation-based clustering of metabolites. Names of all individual samples composing the species clustering are provided in Supplementary Fig. S4. 2-OG, 2-oxoglutarate; 3-PGA, 3-phosphoglycerate; ADPGlc, adenosine 5′-diphosphoglucose; Fru6P, fructose 6-phosphate; Gal1P, galactose 1-phosphate; Glc1P, glucose 1-phosphate; Glc6P, glucose 6-phosphate; Gly3P, glycerine 3-phosphate; Man6P, mannose 6-phosphate; PEP, phospho*enol*pyruvate; Suc6P, sucrose 6′-phosphate; Tre6P, trehalose 6-phosphate; UDPGlc, uridine 5′-diphosphoglucose.

To assess cross-species metabolic trends, we compared *z*-score-transformed metabolite levels across the diel cycle, using the core metabolite dataset with additional metabolites that were below the limit of detection or not measured in only one of the six species analysed (e.g. starch and aconitate in melon, fumarate in strawberry; Fig. 3). Hierarchical clustering of individual time points showed that each species formed a distinct cluster, with subclusters corresponding to daytime and night-time samples of that species (Fig. 3; Supplementary Fig. S5; see also Supplementary Fig. S6 for clustering of species based on separate analyses of day- and night-time samples). Two of the apoplastic loaders (plantago and arabidopsis) clustered together, and were separated from the symplastic loaders (alchemilla, strawberry, melon), whereas the apoplastic loader wheat grouped with the symplastic species. This pattern persisted when only daytime samples were analysed (Supplementary Fig. S6A); however, when only the night-time data were analysed, wheat did not cluster with either group (symplastic or apoplastic phloem loaders) (Supplementary Fig. S6B). Within the symplastic loaders, the passive loaders alchemilla and strawberry formed a separate sub-cluster from melon, the active symplastic RFO loader.

To identify sets of metabolites responsible for this clustering, we first grouped metabolites by Pearson correlation (Fig. 3). Wheat clustered with the symplastic phloem loaders mainly due to elevated levels in metabolite clusters 1a (2-OG, 3-PGA, PEP) and 2a (glucose, fructose, Suc6P, shikimate). Alchemilla and strawberry, which are both members of the *Rosaceae*, displayed near-identical diel metabolite profiles, differing primarily in the following metabolite levels: strawberry had higher levels of 2-OG, PEP, Gly3P, Man6P, Glc6P, and Fru6P, while alchemilla had more shikimate (Fig. 3). Compared with the other species, melon had the highest levels of Glc6P, Fru6P, galactose 1-phosphate (Gal1P), Tre6P, and malate, and elevated glycerate during the day.

To further explore the metabolite relationships within and across species, we conducted pairwise Pearson correlation analyses. This approach enabled us to expand beyond the core metabolite dataset to include all of the metabolites that were measured within a given species (Supplementary Table S1; Supplementary Fig. S7), including metabolites of particular interest in one species, such as sorbitol in plantago.

The correlations were visualized as heatmaps, with metabolites arranged in a fixed order, sorted by metabolic pathway, to facilitate direct comparison across species (Supplementary Fig. S7). All Pearson correlation coefficients and *P*-values are provided in Supplementary Table S2. Visual inspection revealed clear differences in the overall correlation patterns between species and, as a consequence, between individual species and the combined correlation matrix (Supplementary Fig. S7). The positive correlation between sucrose and Tre6P was observed in all six species. This association was not preserved in the combined dataset using only the common metabolites across species (Supplementary Fig. S7), due to the very high Tre6P and highTre6P:sucrose ratios in melon. When melon was excluded, an *r*-value of 0.81 (*P*<2.2×10^−16^) was obtained between Tre6P and sucrose.

We observed some species-specific patterns of correlation in plantago, which transports sorbitol in addition to sucrose. For instance, we found noteworthy differences for Tre6P and sucrose correlations in plantago. Here, the highest and most significant correlations for Tre6P were with sucrose (*r*=0.57) and more strongly with several sugar phosphates including glucose 1,6-bisphosphate, fructose 1,6-bisphosphate, Suc6P, Fru6P, Glc6P (*r*=0.75, 0.74, 0.70, 0.68), and uridine 5′-diphosphoglucose (UDPGlc; *r*=0.56) (all significant) while sucrose correlated the most with Suc6P (*r*=0.81), glucose, and fructose (*r*=0.68, 0.50). The strongest correlation for sorbitol was with Gal1P (*r*=0.50).

### Correlation-based network analyses reveal differences between species in their metabolic networks

**Fig. 4.**
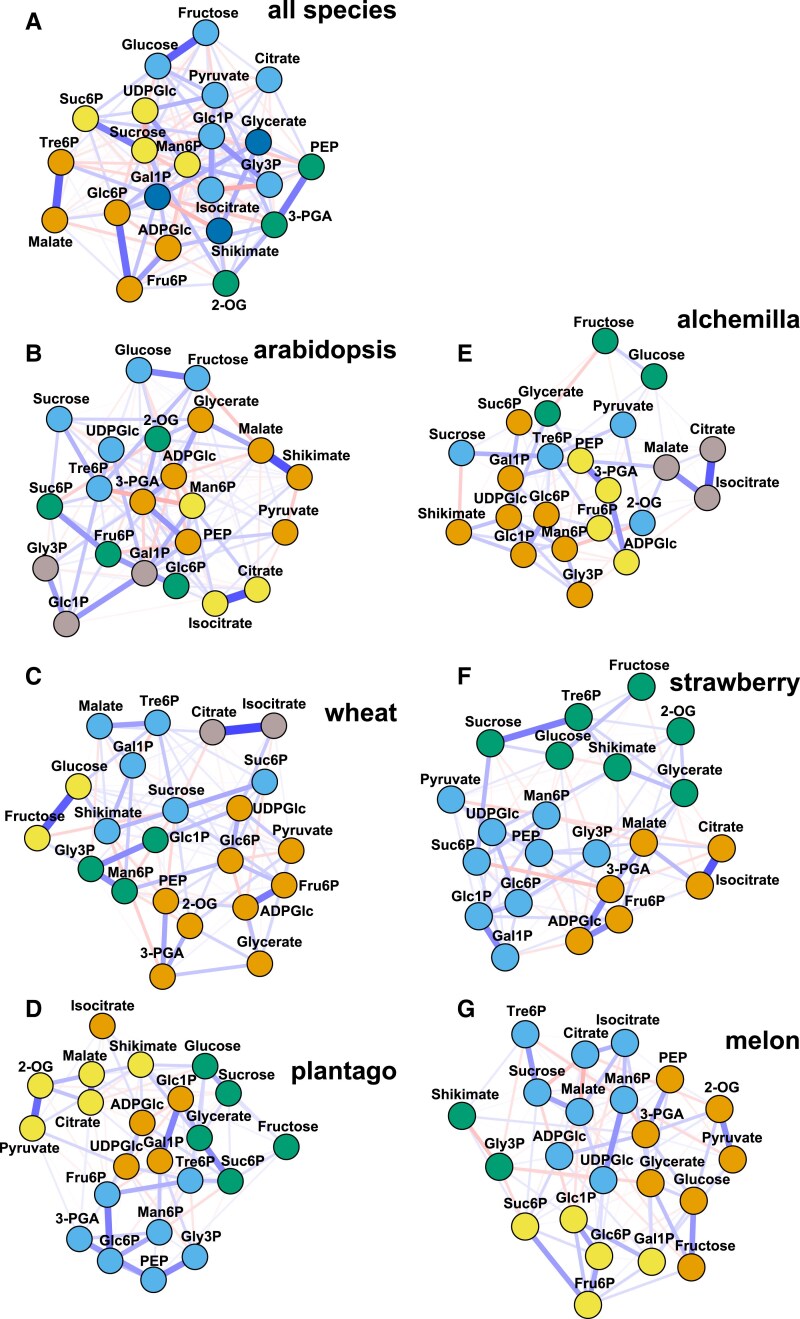
Correlation-based network analysis with community detection of all common metabolites. Associations were detected by Gaussian graphical modelling [either positive (blue) or negative (red)] with the thickness of the edges indicating the strength of the correlation network. A community detected approach was used with metabolites shown in the same colour when they are assigned to the same community. (A) combined dataset of all species, (B) arabidopsis, (C) wheat, (D) plantago, (E) alchemilla, (F) strawberry, and (G) melon. 2-OG, 2-oxoglutarate; 3-PGA, 3-phosphoglycerate; ADPGlc, adenosine 5′-diphosphoglucose; Fru6P, fructose 6-phosphate; Gal1P, galactose 1-phosphate; Glc1P, glucose 1-phosphate; Glc6P, glucose 6-phosphate; Gly3P, glycerine 3-phosphate; Man6P, mannose 6-phosphate; PEP, phospho*enol*pyruvate; Suc6P, sucrose 6′-phosphate; Tre6P, trehalose 6-phosphate; UDPGlc, uridine 5′-diphosphoglucose.

To assess inter-metabolite relationships and, in particular, to compare metabolic network structures across species, we performed network analysis based on Gaussian graphical modelling using diel time-series data from the core 22 metabolites that were measured across all species. Gaussian graphical modelling corresponds to partial correlation analysis for pairs of metabolites while controlling the effect of all other metabolites in the dataset. Correlations between metabolites are represented as edges—positive (blue) or negative (red)—with edge thickness proportional to the correlation strength (Fig. 4). To identify functional modules and hidden relationships within the networks, we applied a community detection algorithm using a greedy optimization of modularity, with metabolites belonging to the same community being colour-coded (Fig. 4).

This integrated network and community detection analysis revealed clear differences in the metabolic networks between individual species (Fig. 4B–G), and, as a result, between any individual species and the combined multi-species network (Fig. 4A). These species-specific patterns underscore the variability in metabolic regulation among species with different phloem loading strategies. Nevertheless, sucrose and Tre6P were consistently connected across five of six individual species networks (Fig. 4; Supplementary Fig. S8), suggesting a robust and conserved relationship between these two metabolites. The only exception was plantago, which mainly transports sorbitol, where the link between Tre6P and sucrose was absent (Fig. 4B–G; Supplementary Fig. S8), although it was detected in paired correlation analysis across the entire diel cycle and the night-time samples (see above). Whereas most of the linked metabolite pairs are in a shared metabolic pathway, Tre6P and sucrose are in separate metabolic pathways implying that the link is the result of signalling. This finding points to a robust relationship between sucrose and Tre6P, independently of differences in the remainder of the metabolic network. The network analyses of the combined dataset further revealed a strong connection between Tre6P and malate (Fig. 4A; Supplementary Fig. S8), a link that was also detected in the wheat- and melon-specific networks (Fig. 4C, G; Supplementary Figs S8C, G).

## Discussion

### The sucrose–trehalose 6-phosphate nexus is conserved in flowering plants with different phloem loading strategies

To assess whether Tre6P functions as a sucrose-specific signal across diverse plant species with different carbon partitioning and translocation strategies, we performed a detailed comparative analysis of the metabolic profiles of source leaves of six species representing the diversity of carbon metabolism and transport in angiosperms. Alchemilla and strawberry maintained very high levels of sucrose during both the day and night (Fig. 1A), consistent with their passive mechanism of symplastic phloem loading. Melon contained substantial levels of raffinose and stachyose (Supplementary Fig. S2), as expected for members of the *Cucurbitaceae* that use the polymer trapping mechanism for active symplastic phloem loading. Sucrose levels rose during the day and then fell at night (Fig. 1A), suggesting that melon leaves also accumulate a substantial pool of sucrose as a transitory carbon store, in addition to the pool of sucrose associated with phloem loading (Fig. 1A). Raffinose and stachyose were at a much lower overall level than sucrose (0.2–0.5 and 1.2–1.6 µmol g^−1^ FW compared with 5–13 µmol g^−1^ FW), consistent with their being synthesized within the phloem complex. Among the apoplastic phloem loaders, arabidopsis and plantago accumulated some sucrose in their leaves during the day, but the levels were much lower than in wheat (Fig. 1A), which is known to store sucrose in preference to starch in its leaves (Trevanion *et al*., 2004). Plantago leaves had high levels of sorbitol (8–18 µmol g^−1^ FW) compared with sucrose (2–4 µmol g^−1^ FW) (Supplementary Fig. S2). These observations were consistent with the reported phloem loading and carbon storage strategies employed by the six species in this study, and the selected species illustrate the diversity of carbon metabolism in angiosperms.

There were striking differences in the levels of Tre6P in the six species, ranging from 0.1–0.3 nmol g^−1^ FW in arabidopsis and plantago to 10–50 nmol g^−1^ FW in melon (Fig. 1A). The other apoplastic phloem loading species, wheat, also had relatively low levels of Tre6P (0.2–0.8 nmol g^−1^ FW), while the two passive symplastic phloem loaders, alchemilla and strawberry, had intermediate levels (1–3 nmol g^−1^ FW) (Fig. 1A). Despite the orders of magnitude differences in absolute levels, Tre6P was significantly correlated with sucrose in each of the six species when the correlation analysis included all samples from the 24 h diel cycle (Fig. 1B; Table 1). The highest correlations were seen in arabidopsis and were also high and statistically significant when the day and night samples were analysed separately (Table 1). The correlations between Tre6P and sucrose were generally weaker in the other species and more dependent on the time of day (Table 1). We also applied network analysis to the data for all of the measured metabolites and this confirmed a sucrose–Tre6P connection in all species except plantago (Fig. 4), where Tre6P showed stronger associations with other sugar phosphates (Supplementary Table S2). Overall, our results show that diel fluctuations in Tre6P levels broadly reflect changes in sucrose levels in leaves of six diverse angiosperm species, consistent with a conserved connection between these metabolites across species with different phloem loading and carbon storage strategies. Most of the linked metabolite pairs identified in our correlation and network analyses are intermediates in the same metabolic pathway, and the link presumably emerges from coordinated changes of intermediate levels when pathway flux changes. However, while Tre6P and sucrose are both synthesized from a common pool of hexose phosphates and UDPGlc, they lie on separate metabolic pathways that compete with each other for substrates. From the perspective of pathway topology, we might therefore even expect a negative correlation between Tre6P and sucrose. The observed positive correlation between these metabolites implies that the link is the result of signalling. In the following section we discuss why the very strong sucrose–Tre6P correlation seen in arabidopsis leaves might be less apparent in some other species, and whether these differences can inform us about the functions of Tre6P in leaves.

### The potential impact of intra- and intercellular compartmentation of sucrose on its relationship with trehalose 6-phosphate

The Tre6P-synthesizing enzyme AtTPS1 is largely expressed in the vasculature in arabidopsis (see Introduction; Fichtner *et al*., 2020). Non-aqueous fractionation experiments indicated that Tre6P is located primarily outside the chloroplasts and vacuoles in arabidopsis leaf cells (Martins *et al*., 2013), but this method does not distinguish the cytosol and nucleus (Arrivault *et al*., 2014). AtTPS1 is present in both the cytosol and nucleus, indicating that Tre6P is synthesized in these compartments and can probably move freely between them via pores in the nuclear membrane (Fichtner *et al*., 2020). The inferred location of Tre6P synthesis in and around the vascular tissue and the information about subcellular fractionation suggest that Tre6P is linked to the metabolically active pool of sucrose associated with phloem loading and transport. Arabidopsis stores very little sucrose in its leaf vacuoles, so most of the sucrose in the rosette will be in this metabolically active transport pool, which could explain why we consistently observe very strong correlations between Tre6P and sucrose when measured in whole rosettes of this species (Lunn *et al*., 2006; Martins *et al*., 2013; Fichtner *et al*., 2020, 2021a, b).

In contrast, wheat stores substantial amounts of sucrose in its leaves during the day (Fig. 1A), mainly in the vacuoles. Therefore, if Tre6P primarily reflects the metabolically active transport pool of sucrose, the correlation between sucrose and Tre6P in whole-leaf measurements would appear to be weaker in wheat, due to dilution by the less metabolically active storage pool of sucrose in the vacuoles, which is supported by our findings (Fig. 1B; Table 1; Supplementary Table S2).

In passive symplastic phloem loaders, the mesophyll cells where sucrose is made are in symplastic connection with the companion cells and sieve elements of the phloem, and sucrose must be maintained at high concentrations in all of these cells to generate the hydrostatic pressure required for phloem transport to sink organs. In arabidopsis leaves, we can infer from the localization of AtTPS1 that there is limited synthesis of Tre6P in the mesophyll cells where sucrose is made, but it is synthesized in cells associated with the vascular bundles that are symplastically connected with the mesophyll (Fichtner *et al*., 2020). There is evidence that this pattern is conserved in other flowering plants, including symplastic phloem loaders, as recent single-cell RNA sequencing data from wheat and *P. mume* have shown that *TPS1* transcripts are specifically expressed in phloem-related cell types (Zhang *et al*., 2023; Guo *et al*., 2024). If Tre6P is also synthesized in the phloem cells in alchemilla and strawberry, the levels of Tre6P might specifically reflect the sucrose concentrations in those cells, but due to the symplastic connectivity they are also likely to reflect the levels of sucrose in the mesophyll cells as well. Therefore, a correlation between Tre6P and sucrose might be expected even from whole-leaf metabolite measurements.

However, we observed weaker sucrose–Tre6P correlations in alchemilla and strawberry than in arabidopsis (Fig. 1B; Table 1). It should be noted that the sucrose levels in alchemilla and strawberry leaves fluctuated within a very narrow range during the diel cycle, which could limit the power of the correlation analysis (Fig. 1A; Table 1).

In plantago, Tre6P and sucrose were significantly correlated during the 24 h diel cycle and during the night-time (Table 1; Supplementary Table S2); however, they were not found to be in the same metabolic network community in the Gaussian graphical model network analysis suggesting a more complex interaction between Tre6P and sucrose in plantago compared with the other species. In plantago leaves, *PmTPS1* is expressed predominantly in the vasculature (Huang *et al*., 2019), indicating that this is the main site for Tre6P synthesis. The diel fluctuations in plantago leaf sucrose levels, particularly at the beginning of the day and night (Fig. 1A), suggest that there is a substantial storage pool of sucrose in this species. As discussed above, if Tre6P is primarily linked to the cytosolic pool of sucrose that is available for export, large storage pools of sucrose in the vacuoles can partially mask this link, leading to weaker correlations between sucrose and Tre6P based on whole-leaf metabolite measurements. Another potential explanation stems from the specialized carbon metabolism in plantago leaves, which are unusual in having high levels of sorbitol as well as sucrose. Like sucrose, sorbitol is synthesized from the hexose phosphate pool in the cytosol. In plantago, Tre6P showed very strong correlations with a set of sugar phosphates (Supplementary Table S2) that are precursors for both sorbitol (Pleyerová *et al*., 2022) and sucrose biosynthesis (Lunn, 2016). This raises an intriguing possibility that the sucrose–Tre6P nexus has been adapted in plantago to connect Tre6P to sorbitol and sucrose production.

### Trehalose 6-phosphate is linked to malate in cross-species analyses

In addition to the well-established link with sucrose, our metabolic network analysis revealed a link between Tre6P and malate (Fig. 4A, Supplementary Fig. S8A). Malate has many functions in plants; it is an intermediate in the TCA cycle in the mitochondria and plays important roles in redox balancing between chloroplasts, cytosol, and mitochondria (the malate valve) and pH homeostasis, and can also be a significant carbon reserve (Dao *et al*., 2022). Previously, it was observed that Tre6P regulates the anaplerotic flux of carbon into malate in leaves via post-translational regulation of PEPC (Figueroa *et al*., 2016). Tre6P has also been implicated in regulation of PEPC in roots, where malate is exuded into the soil to facilitate uptake of nutrients (Shane *et al*., 2016). In the chlorophyte alga *Chlamydomonas reinhardtii*, a *tpp* mutant that accumulated high levels of Tre6P also showed an increase in TCA cycle intermediates, especially malate and fumarate (Youssef *et al*., 2023). These findings further support the hypothesis that the activation of carbon flux into the TCA cycle by Tre6P is a conserved signalling pathway in plants. It is possible that this role of Tre6P in carbon metabolism represents an ancient function that predates the evolution of land plants. However, as the link between malate and Tre6P was not consistently identified across all species, further research must demonstrate if Tre6P and malate are indeed consistently connected, or if the link might be species specific and/or context dependent.

In conclusion, the sucrose–Tre6P nexus model postulates a dual function for Tre6P as a signal and negative feedback regulator of sucrose levels. In arabidopsis leaves, the strong positive correlation between sucrose and Tre6P levels during the diel cycle is an emergent property of the nexus. Our results showed that this basic relationship is conserved in five other species, and to a certain extent in a sixth species, which either use different mechanisms for phloem loading or transport a mixture of sucrose and other carbohydrates, and which accumulate different carbohydrates in their leaves during the day to support night-time respiration and growth. Furthermore, our results reveal a frequent link between Tre6P and malate, which suggests that the regulation of PEPC and anaplerotic flux by Tre6P seen in arabidopsis leaves (Figueroa *et al*., 2016) also occurs in other species. Thus, this study provides evidence that the sucrose–Tre6P nexus does operate in a diverse range of angiosperms, and that some of the component molecular mechanisms elucidated in arabidopsis are conserved in the other species. Nevertheless, our results also point to adaptations of the nexus to meet the individual needs of each species, reflecting differences in their carbon metabolism and transport. This echoes the flexibility of the sucrose–Tre6P nexus that is seen within an individual species, where it is adapted to meet the specific needs of different organs, developmental stages and environmental conditions (Figueroa and Lunn, 2016).

## Supplementary Material

eraf401_Supplementary_Data

## Data Availability

The primary data supporting this study are available in Supplementary Table S1, and our FAIR data publication can be found under DOI: https://doi.org/10.60534/bj5s3-wvh69.

## Supplementary data

The following supplementary data are available at *JXB* online.

Fig. S1. Phylogenetic distribution based on APG IV (Angiosperm Phylogeny Group IV, 2016) of the plant species used in this study.

Fig. S2. Relationship between phloem-transported sugars and Tre6P in melon and plantago.

Fig. S3. Tre6P:sucrose ratios in different species.

Fig. S4. Principal component analysis (PCA) of metabolite data from all datasets showing the individual time points.

Fig. S5. Clustering of species and all time points.

Fig. S6. Cross-species comparison of metabolite levels via hierarchical clustering analysis of all common metabolites for daytime and night-time.

Fig. S7. Pairwise correlations between metabolites in different metabolite classes with names of all metabolites.

Fig. S8. Correlation-based network analysis with community detection highlighting the connections between Tre6P, sucrose, and malate.

Table S1. Raw data used in this study.

Table S2. Statistics of the correlation analyses of this study.
