# Supplementary material for: The sucrose–trehalose 6-phosphate nexus is conserved in flowering plants with different phloem loading and carbon storage strategies
Source: J Exp Bot. 2025 Oct 23;77(2):578–91. doi: 10.1093/jxb/eraf401 (PMC12794214; doi:10.1093/jxb/eraf401)
Supplement: eraf401_Supplementary_Data [file eraf401_supplementary_data.zip › jexbot316141-file001.pdf]

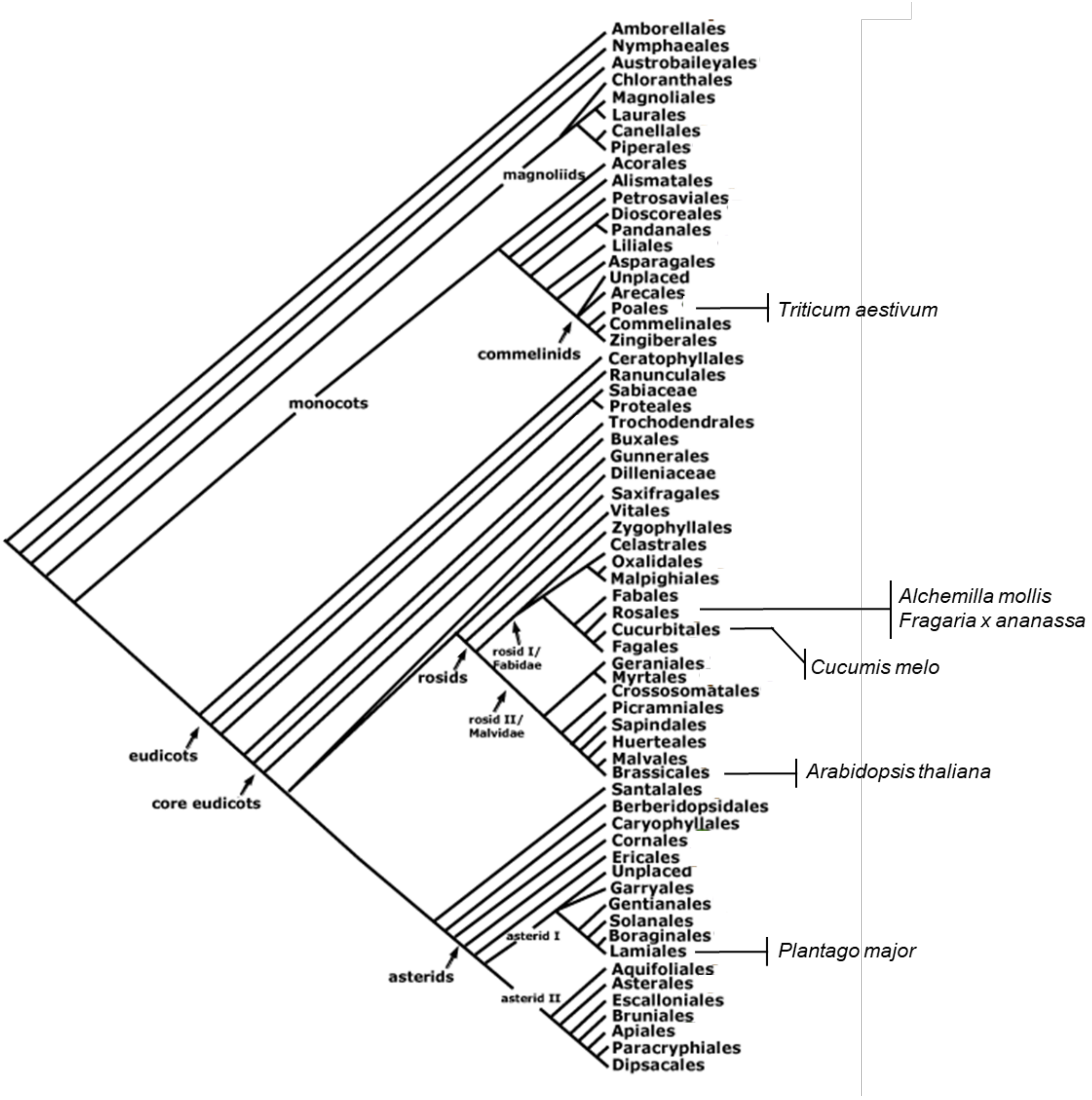

Supplementary Fig. S1. Phylogenetic distribution based on APG IV (Angiosperm Phylogeny Group IV, 2016) of the plant species used in this study. Adapted from Stevens, P. F. (2001 onwards). Angiosperm Phylogeny Website. Version 14, July 2017; http://www.mobot.org/MOBOT/research/APweb/

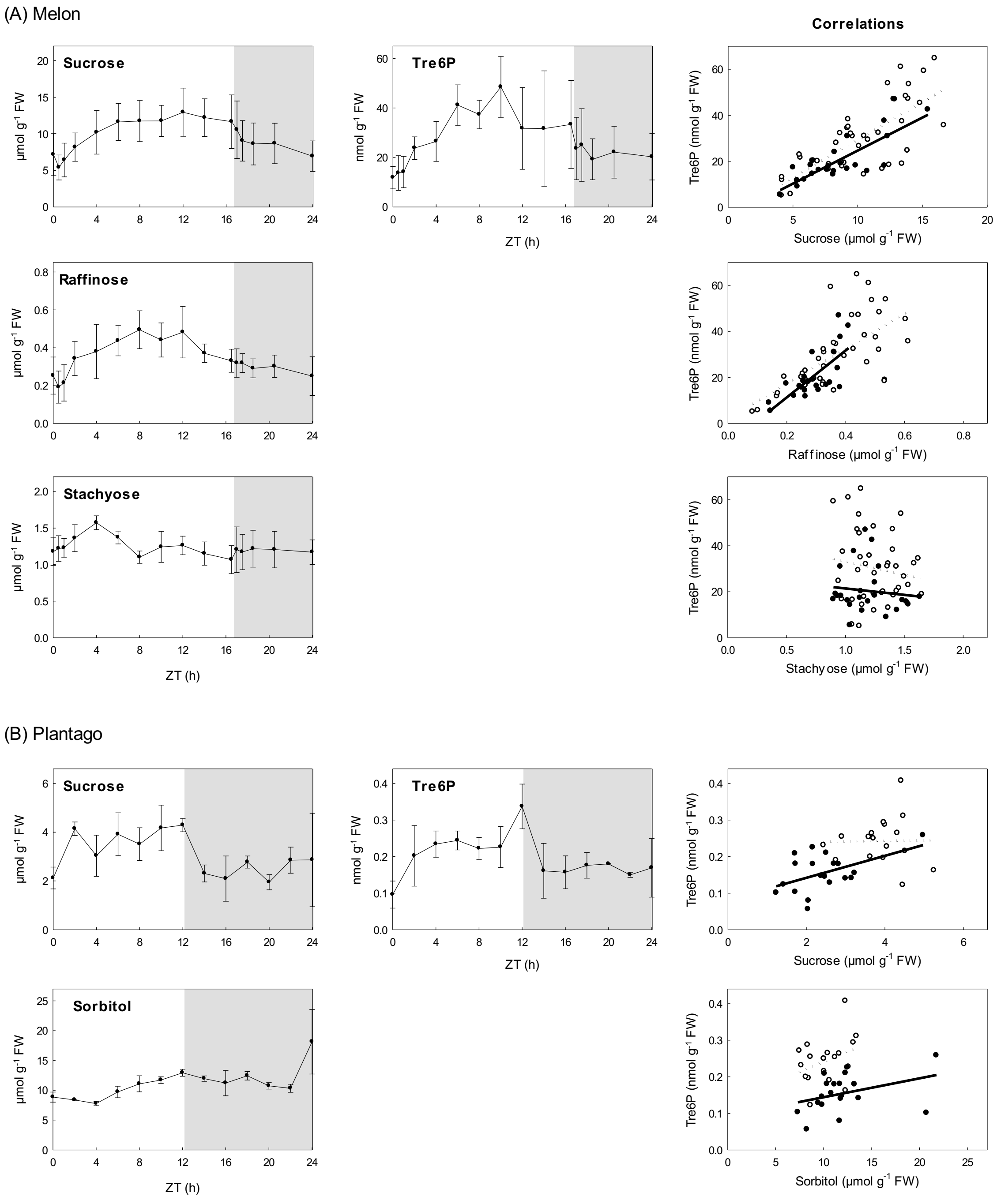

Supplementary Fig. S2. **Relationship between phloem-transported sugars and Tre6P in melon and plantago.** The left and centre columns show diel changes of **(A)** sucrose, raffinose, stachyose and Tre6P in melon (*Cucumis melo*) leaves (mean ± S.D., *n* =4) and **(B)** sucrose, sorbitol and Tre6P in plantago (*Plantago major*) leaves (mean ± S.D., *n* = 2-3). The right-hand columns show the correlations between each sugar and Tre6P in the respective species, with linear regression lines for the daytime (open symbols, dotted line) and night-time samples (closed symbols, solid line). The sucrose and Tre6P data for each species are the same as those in Fig. 1 and are copied here for ease of reference. ZT, zeitgeber time (time after dawn). The original data are in Supplementary Dataset S1.

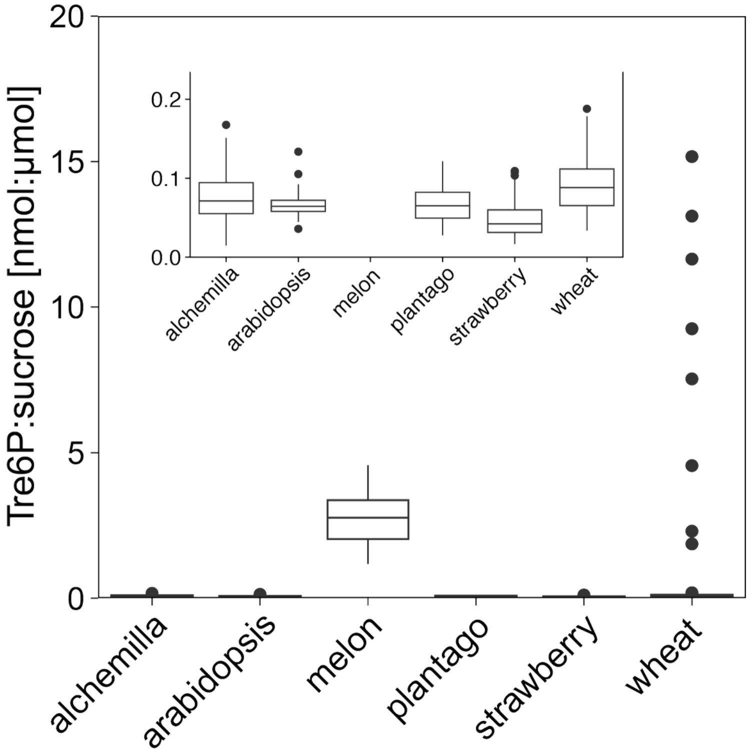

Supplementary Figure S3. Tre6P:sucrose ratios in different species. The Tre6P:sucrose ratio was calculated for each individual sample from the diel cycle experiments shown in Fig. 1. The values for each species are presented as boxplots showing the first quartile, median and third quartile, with the whiskers showing 1.5x the inter-quartile range. Individual outliers are shown separately. The inset panel shows the species with low Tre6P:sucrose ratios on a magnified scale. The number of samples for each species was: alchemilla n = 84 arabidopsis n= 92, melon n = 64, plantago n = 37, strawberry n = 85, wheat n = 68. The original data are in Supplementary Dataset S1.

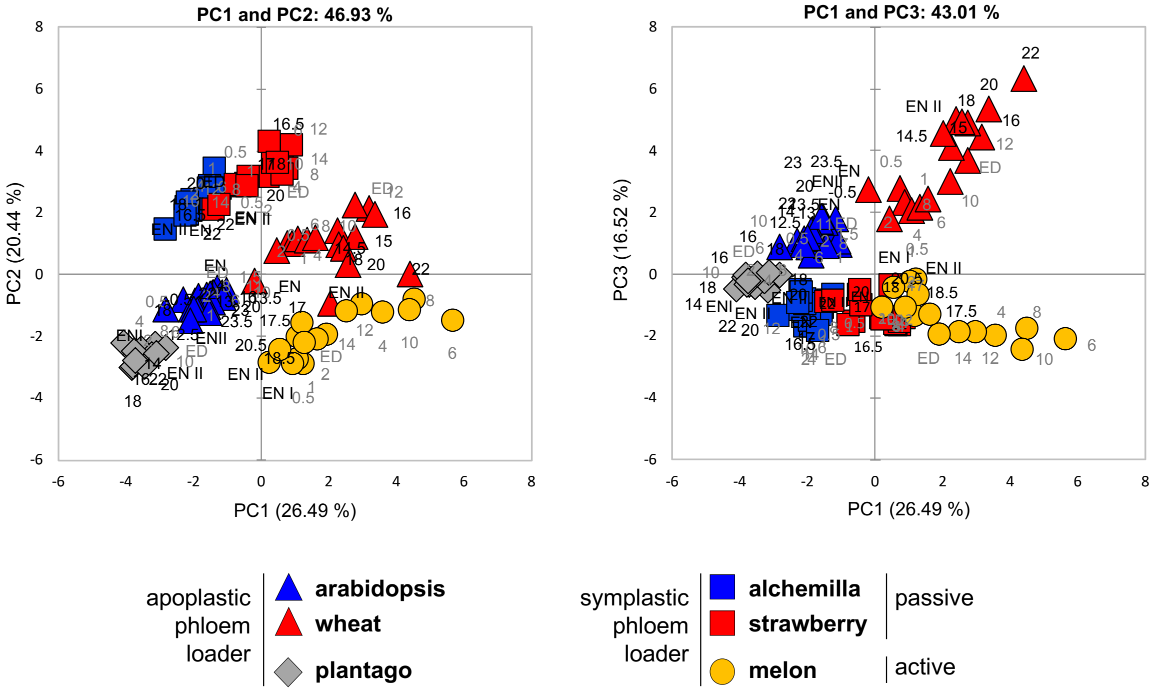

Supplementary Fig. S4. Principal component analysis (PCA) of metabolite data from all data sets showing the individual time points. PCA of metabolite data from arabidopsis (blue triangle) and plantago (grey diamond) plants grown in controlled environment chambers with a 12 h photoperiod, wheat (red triangle), alchemilla (blue square) and strawberry (red square) plants grown in controlled environment chambers with a 14 h, 16 h and 16 h photoperiod, respectively, and melon (yellow circle) plants grown in a naturally illuminated greenhouse in a 16.5 h natural photoperiod. The percentages of total variance represented by principal component 1 (PC1), PC2 and PC3 are shown in parentheses. Numbers indicate the time of harvest in hours after dawn (zeitgeber time, ZT); ED, end of day (ZT12); EN I end of preceding night (ZT0); EN II, end of night (ZT24). Day harvested samples are indicated in grey; night harvested samples are indicated in dark. Only metabolites measured in all data sets were included in the PCAs.

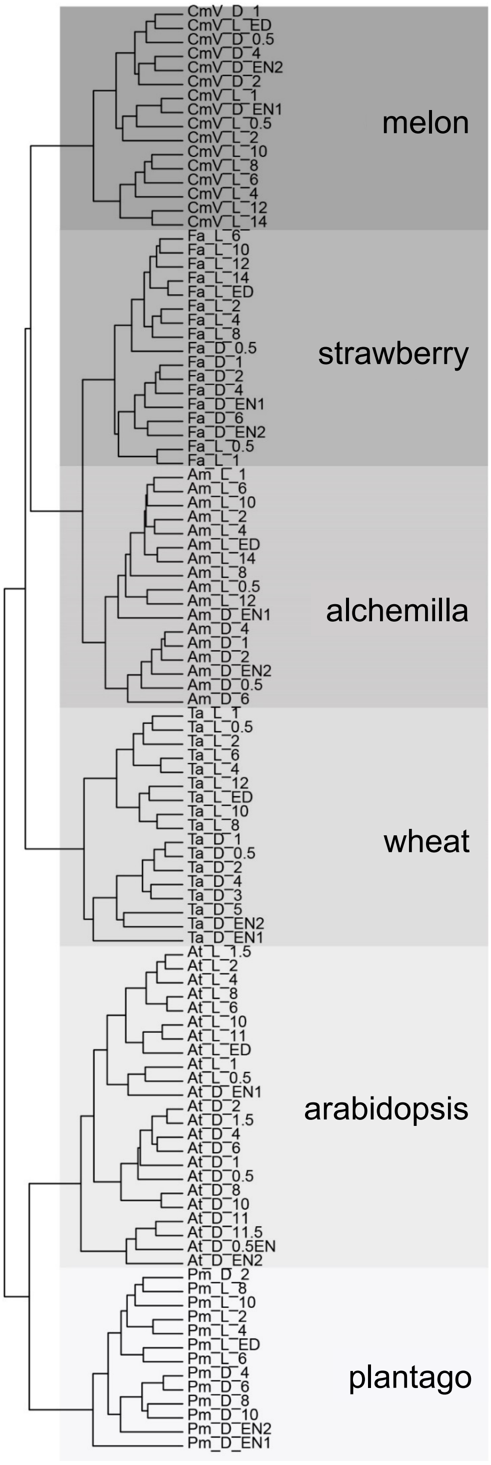

Supplementary Fig. S5. Clustering of species and all time points. Dendrograms represent clusters based on a canberra distance matrix with mcquitty-based clustering. Abbreviations of the scientific names are used. Letters L and D indicate samples harvested in the light and in the dark, respectively. Numbers indicate the time of harvest in hours after dawn (zeitgeber time, ZT); ED, end of day (ZT12 in arabidopsis and plantago; ZT14 in wheat; ZT16 in alchemilla and strawberry; ZT16.5 in melon); EN1 end of preceding night (ZT0); EN2, end of night (ZT24).

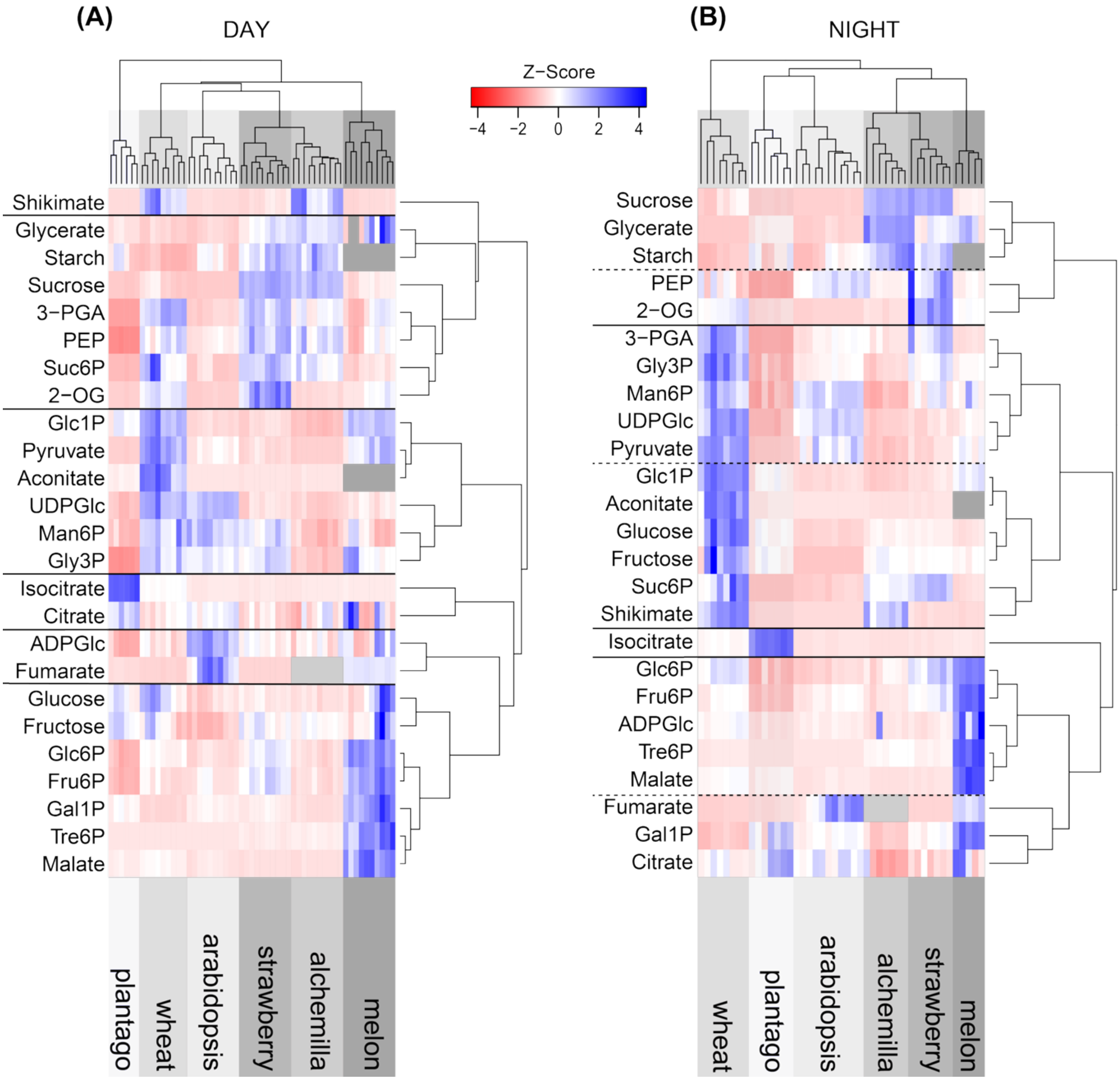

Supplementary Fig. S6. Cross-species comparison of metabolite levels via hierarchical clustering analysis of all common metabolites for (A) daytime and (B) night-time. *Z*-scores of the mean are presented for each individual metabolite, with high and low Z-scores shown in blue or red, respectively. Dashed and solid lines highlight clusters among metabolites. Dendrograms represent clusters based on a canberra distance matrix with mcquitty-based clustering of species and correlation-based clustering of metabolites. 2-OG, 2-oxoglutarate; 3-PGA, 3-phosphoglycerate; ADPGlc, adenosine 5′-diphosphoglucose; Fru6P, fructose 6-phosphate; Gal1P, galactose 1-phosphate; Glc1P, glucose 1-phosphate; Glc6P, glucose 6-phosphate; Gly3P, glycerine 3-phosphate; Man6P, mannose 6-phosphate; PEP, phospho*enol*pyruvate; Suc6P, sucrose 6′-phosphate; Tre6P, trehalose 6-phosphate; UDPGlc, uridine 5′-diphosphoglucose.

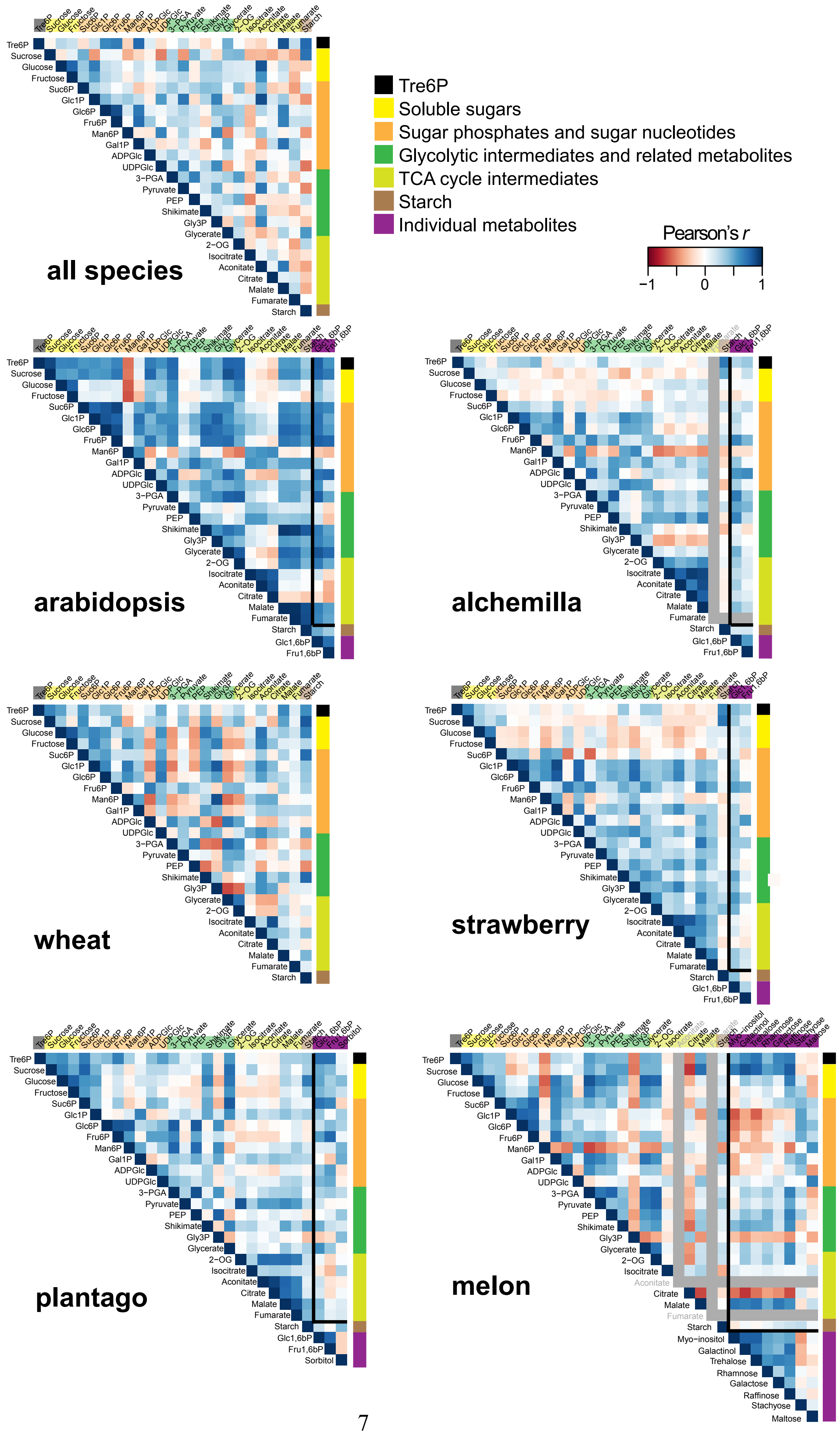
Tre6P
Soluble sugars
Sugar phosphates and sugar nucleotides
Glycolytic intermediates and related metabolites
TCA cycle intermediates
Starch
Individual metabolites
Pearson's r
−1
0
1
all species
arabidopsis
alchemilla
wheat
strawberry
plantago
melon
Tre6P
Sucrose
Glucose
Fructose
Suc6P
Glc1P
Glc6P
Fru6P
Man6P
Gal1P
ADPGlc
UDPGlc
3−PGA
Pyruvate
PEP
Shikimate
Gly3P
Glycerate
2−OG
Isocitrate
Aconitate
Citrate
Malate
Fumarate
Starch
Glc1,6bP
Fru1,6bP
Sorbitol
Myo−inositol
Galactinol
Trehalose
Rhamnose
Galactose
Raffinose
Stachyose
Maltose

Supplementary Fig. S7. Pairwise correlations between metabolites in different metabolite classes with names of all metabolites. The plots show correlations between individual metabolite pairs, with the metabolites organised in metabolite classes indicated by the coloured bars at the side of the heat maps. Each square represents the correlation between the metabolite heading the column with the metabolite heading the row. In cases where a metabolite is absent in two or more species, it is assigned to the class 'individual metabolites' delimited by the black lines. All other metabolites are in the same order in all species. Metabolites that are missing in only one species are kept in the same order and presented in grey. The Pearson's coefficient values (r) is indicated with high values shown in blue (representing a strong positive correlation) and low values shown in red (representing a strong negative correlation).

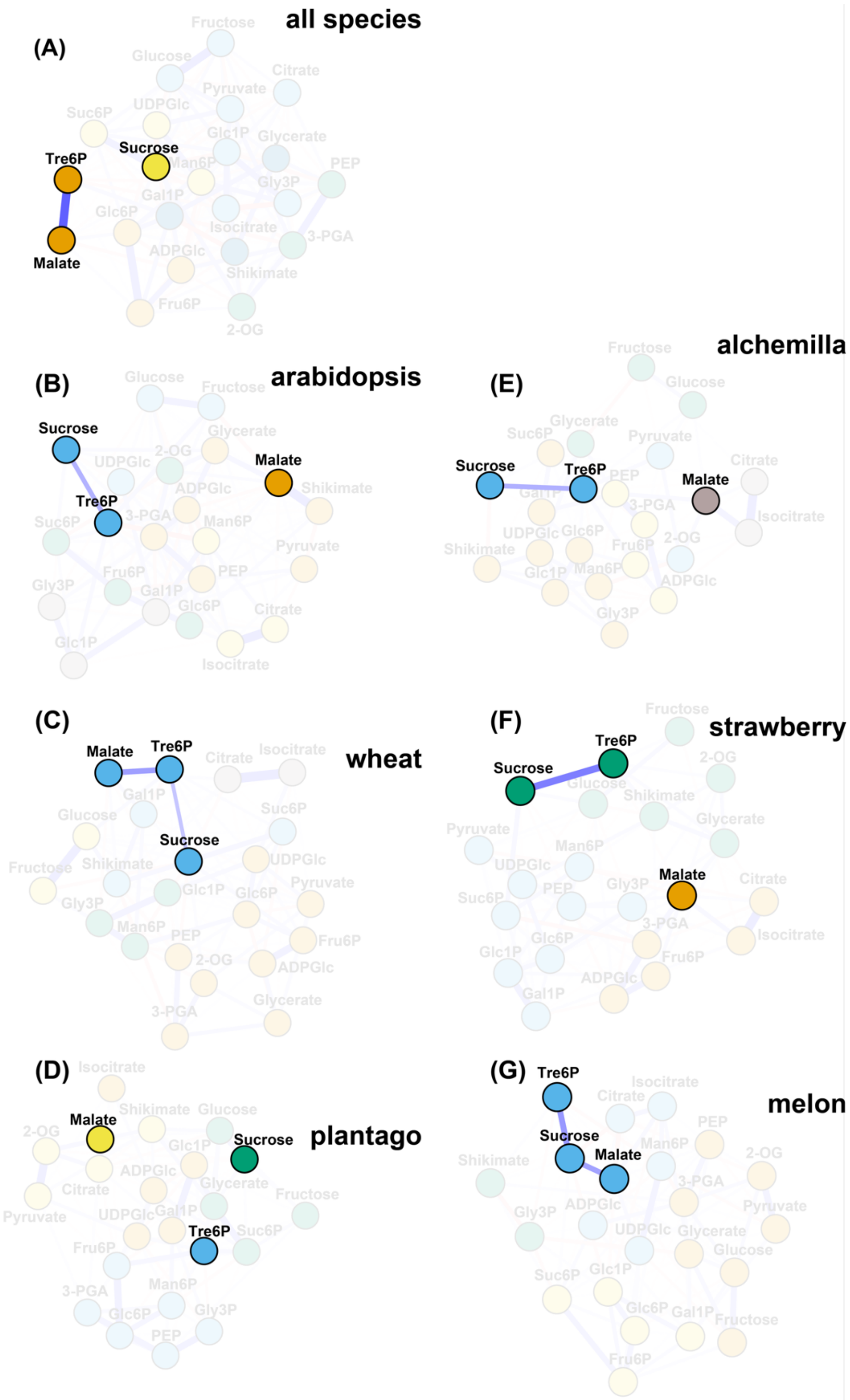

Fig. S8. Correlation-based network analysis with community detection highlighting the connections between Tre6P, sucrose and malate. Associations were detected by graphical gaussian modelling (GGM) from Fig. 4 with Tre6P, sucrose, and malate highlighted. **A,** combined data set of all species, **B**, arabidopsis, **C**, wheat, **D**, plantago, **E,** alchemilla, **F,** strawberry, and **G,** melon.
